# Molecular Diversity of Fungal Phylotypes Co-Amplified Alongside Nematodes from Coastal and Deep-Sea Marine Environments

**DOI:** 10.1371/journal.pone.0026445

**Published:** 2011-10-26

**Authors:** Punyasloke Bhadury, Holly Bik, John D. Lambshead, Melanie C. Austen, Gary R. Smerdon, Alex D. Rogers

**Affiliations:** 1 Integrative Taxonomy and Microbial Ecology Research Group, Department of Biological Sciences, Indian Institute of Science Education and Research-Kolkata, Kolkata, West Bengal, India; 2 Hubbard Center for Genome Studies, University of New Hampshire, Durham, New Hampshire, United States of America; 3 School of Ocean and Earth Sciences, University of Southampton, Southampton, United Kingdom; 4 Plymouth Marine Laboratory, Plymouth, United Kingdom; 5 Department of Zoology, University of Oxford, Oxford, United Kingdom; J. Craig Venter Institute, United States of America

## Abstract

Nematodes and fungi are both ubiquitous in marine environments, yet few studies have investigated relationships between these two groups. Microbial species share many well-documented interactions with both free-living and parasitic nematode species, and limited data from previous studies have suggested ecological associations between fungi and nematodes in benthic marine habitats. This study aimed to further document the taxonomy and distribution of fungal taxa often co-amplified from nematode specimens. A total of 15 fungal 18S rRNA phylotypes were isolated from nematode specimens representing both deep-sea and shallow water habitats; all fungal isolates displayed high pairwise sequence identities with published data in Genbank (99–100%) and unpublished high-throughput 454 environmental datasets (>95%). BLAST matches indicate marine fungal sequences amplified in this study broadly represent taxa within the phyla Ascomycota and Basidiomycota, and several phylotypes showed robust groupings with known taxa in phylogenetic topologies. In addition, some fungal phylotypes appeared to be present in disparate geographic habitats, suggesting cosmopolitan distributions or closely related species complexes in at least some marine fungi. The present study was only able to isolate fungal DNA from a restricted set of nematode taxa; further work is needed to fully investigate the taxonomic scope and function of nematode-fungal interactions.

## Introduction

In benthic marine environments, free-living nematodes often outnumber other groups in terms of diversity and abundance and are known to play key roles in ecosystem processes [1], [2]. Marine fungi represent another ecologically important group amongst benthic organisms, acting as key intermediates of energy flow from detritus to higher trophic levels in marine ecosystems [3], [4], [5]. The ecological importance of filamentous fungi in marine systems is underestimated; these organisms represent a diverse range of saprobes, pathogens and symbionts forming an integral part of coastal and deep-sea environments [6], [7].

Free-living nematodes are also known to harbor microbes on their body surface, dominated mostly by symbiotic bacterial phylotypes [8], [9]. In addition, it has been suggested that nematode species may actively ‘farm’ microfaunal species using mucus-agglutinated detritus to provide nutrition [10]; there is even evidence to suggest that such detrital agglutinations facilitate commensalism and shared metabolic pathways between nematodes and microbes [11]. Marine fungal species have been reported on a variety of substrata including decaying woods, leaves, seaweeds, seagrasses, and other calcareous and chitinous surfaces [12], [13]. Although less research has been conducted on nematode-fungal relationships, some association between fungi and soil nematodes been also reported in the literature [14], and it is likely that similar relationships exist amongst marine species.

In a recent study we provided preliminary evidence of possible ecological association between fungi and free-living marine nematodes in the coastal sedimentary environments from southwest England based on 18S rRNA PCR-DGGE approach [15]. Here we show further evidence of such association from another offshore site in southwest England and from a deep-sea site in the Southern Indian Ocean when nematodes were targeted with nematode specific 18S rRNA primers. In addition, we also provide evidence that some of these fungal 18S rRNA phylotypes are ubiquitous and may have a broad geographic distribution.

## Results

No amplification of common airborne fungal spores was detected during the course of the study, confirmed by PCR control reactions. In total, 30 nematode specimens from Rame Head (RH) were identified based on morphological characters and subsequently amplified using previously reported nematode 18S rRNA primers (SSU_F04 and SSU_R22). Out of 30 nematodes, 12 co-amplified fungal 18S rRNA amplicons in addition to nematode 18S rRNA sequences. Three different fungal clones were detected alongside Rame Head nematodes: RH1 (three sequences), RH2 (five sequences) and RH3 (four sequences). The fungal sequences from Rame Head consisted of a 355 to 400 bp fragment from the extreme 5′ end of the 18S rRNA. According to BLAST matches (Blastn), fungal sequences showed 98–100% identity with other published fungal 18S sequences in GenBank and >97% identity to unpublished high-throughput environmental sequence data obtained from marine sediments (Table 1). The sequences from fungal clone types RH1, RH2 and RH3 were further examined through BLAST search. The highest matches for RH1 were to an uncultured marine ascomycete 18S rRNA clone PRTBE7395 (Acc No HM799862) amplified from ocean water collected from 6000 depth within the Puerto Rico Trench, with the next closest match representing an uncultured fungal partial 18S rRNA sequence JCF1 (Acc No AJ965493); these two GenBank sequences are perhaps closely related members of the Chaetothyriales (Herpotrichiellaceae). RH1 sequences were thus further aligned against the five best matches from this fungal order, including additional genera within the Chaetothyriales and representative sequences of the other related fungal orders that were listed amongst the 50 best BLAST matches. The best BLAST match to RH2 sequences was an uncultured marine fungus clone FAS_14 (Acc No GQ120110) reported from the oxygen depleted sediments of the Arabian Sea, followed by another uncultured fungus clone (Acc No AJ965494) reported previously from the waters of Southwest England. For RH3, the highest match was to an uncultured eukaryote 18S rRNA clone (Acc No AB510380) from Antarctic lake sediments followed by an uncultured Trichocomaceae clone (Acc No EU085016) isolated from the Hawaiian marine sponge *Suberites zeteki*.

**Table 1:** Fungal phylotypes recovered in this study, detailing best BLAST matches to unpublished 454 data from marine sediments [19] and top-scoring hit amongst published sequences in GenBank.

				Marine 454 Data*				Top Scoring BLAST Mach in GenBank				
Fungal Sequence	Primers	Length	Nematode taxa	SequenceIdentity	Gaps	E-value	Query coverage	Sequence Identity	Gaps	E-value	Query coverage	Taxonomic Identity
DS1	F04/R22	356 bp	*Southerniella* sp.	100%	0/385	0.0	99%	100%	0/390	0.0	100%	Uncultured fungus clone(Acc GU072590)
DS2	F04/R22	404 bp	*Halalaimus* sp*Desmodora* sp.	98%	1/399	0.0	99%	100%	0/404	0.0	100%	Uncultured Agaricomycotina clone(Acc EU647009)
DS3	F04/R22	399 bp	*Metadasynemella* sp.*Chromaspirinia* sp.*Amphimonhystrella* sp.	98%	3/395	0.0	99%	99%	0/399	0.0	99%	*Seiridium* sp(Acc AF346558)
DS4	F04/R22	380 bp	*Campylaimus* sp.	95%	5/399	0.0	99%	99%	0/402	0.0	100%	*Cryptococcus* sp.(Acc DQ645520)
DS5	F04/R22	397 bp	*Desmoscolex* sp.	99%	0/392	0.0	99%	99%	0/397	0.0	99%	Fungal sp.(Acc GQ120167)
DS6	F04/R22	399 bp	*Daptonema* sp.	99%	1/395	0.0	99%	100%	0/399	0.0	100%	Uncultured ascomycete clone(Acc EU409874)
DS7	F04/R22	390 bp	*Molgolaimus* sp.	100%	0/385	0.0	99%	100%	0/390	0.0	100%	Uncultured fungus isolate(Acc FJ785876)
DS8	F04/R22	404 bp	*Desmodora* sp.	98%	1/399	0.0	99%	100%	0/404	0.0	100%	Uncultured Agaricomycotina clone (Acc EU647009)
RH1	F04/R22	397 bp	*Daptonema* sp.*Daptonema normandicum*	100%	0/392	0.0	99%	100%	0/397	0.0	100%	Uncultured fungus(Acc AJ965493)
RH2	F04/R22	398 bp	*Terschellingia* sp.*Terschellingia longicaudata* *Parodontophora* sp.*Viscosia viscosa* *Sabatieria pulchra*	97%	2/394	0.0	99%	99%	0/398	0.0	100%	Uncultured marine fungus clone(Acc GQ120110)
RH3	F04/R22	390 bp	*Viscosia viscosa* *Sabatieria pulchra*	99%	0/385	0.0	99%	99%	0/390	0.0	100%	Uncultured eukaryote(Acc AB510380)
D9	F22/R24	425 bp	Ceramonematidae sp.	N/A	N/A	N/A	N/A	100%	0/425	0.0	100%	*Monascostroma innumerosum*(Acc GU296179)
D10	F22/R26	523 bp	*Acantholaimus* sp.	N/A	N/A	N/A	N/A	99%	0/522	0.0	99%	*Vuilleminia comedens* strain T-583 (Acc AF518594)
D11	F24/R13	542 bp	*Diplopeltula* sp.	N/A	N/A	N/A	N/A	99%	0/542	0.0	99%	Uncultured fungus clone(Acc AB469139)
D12	F24/R13	478 bp	*Desmoscolex* sp.	N/A	N/A	N/A	N/A	100%	0/478	0.0	100%	Uncultured eukaryote clone(Acc EU682622)

Some 18S regions were not represented in 454 datasets (denoted by N/A).

In total, 40 deep-sea nematode specimens were morphologically identified down to genus level and subsequently amplified using all four sets of nematode 18S primers. Fungal 18S rRNA sequences were detected alongside 18S rRNA amplicons from fifteen deep-sea nematodes in this study. Eight fungi-like phylotypes were detected alongside 18S rRNA amplicons from 11 nematode specimens when targeted with SSU_F04-SSU_R22, denoted as phylotypes DS1, DS2, DS3, DS4, DS5, DS6, DS7 and DS8. The fungal sequences varied between 347–404 bp in length and showed 99–100% identities with published fungal 18S sequences in GenBank and >95% identity to an unpublished high-throughput environmental dataset (Table 1). In addition, shallow-water fungal phylotype DS1 showed 99% pairwise identity to sequence RH3 obtained from shallow-water sediments; DS2 and DS8 were also observed to be identical sequences. Four other fungal 18S rRNA sequence types (DS9 to DS12) were detected in four deep-sea nematodes using a number of other 18S primer sets (Table 1). All these sequences showed 99-100% identity with published fungal 18S sequences based on BLASTn searches. The fungal sequences co-amplified from deep-sea nematodes could be broadly classified under Ascomycota and Basidiomycota fungal groupings based on BLAST matches.

**Figure 1:**
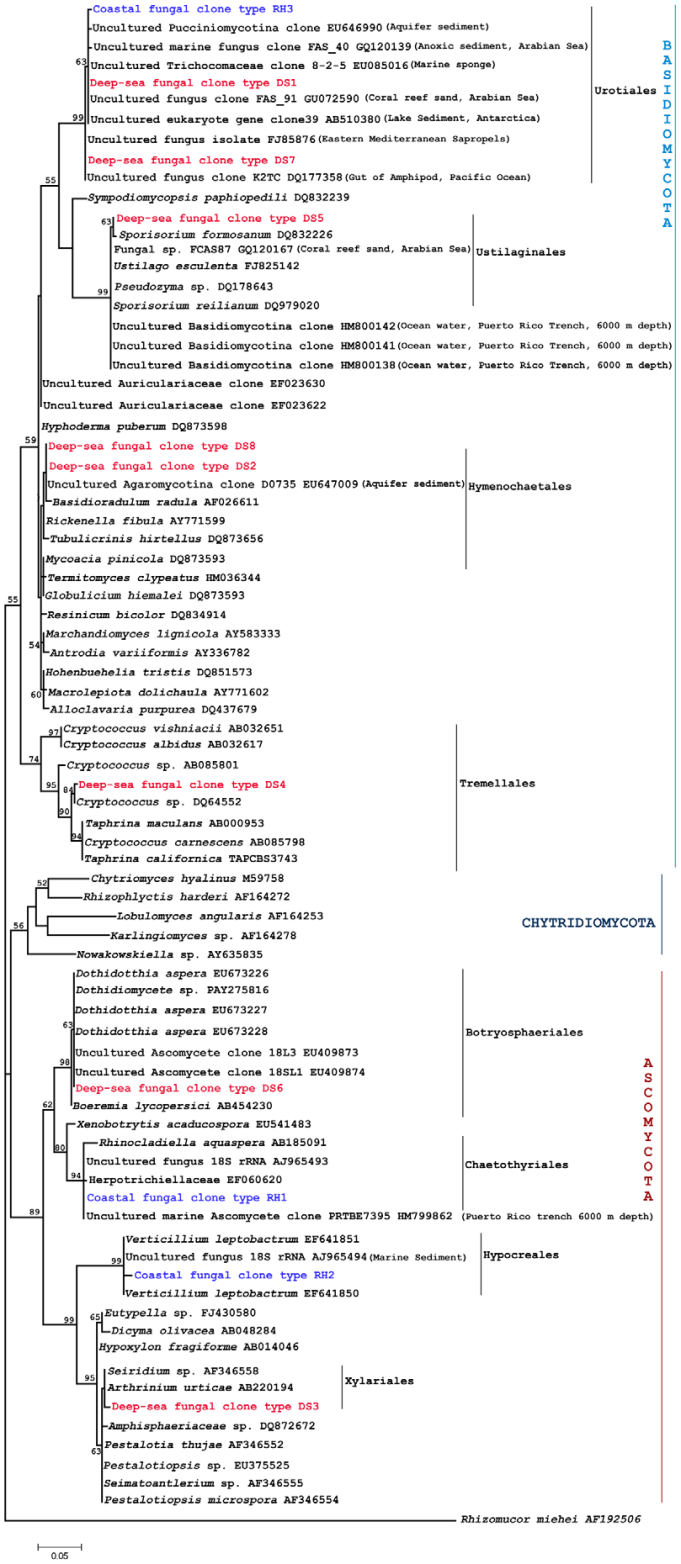
Maximum likelihood tree with bootstrap values constructed using fungal 18S rRNA sequences generated from this study and corresponding BLAST matches with highly identity scores recovered from GenBank and EMBL as well as published cultured fungal 18S rRNA sequences. Bootstrap values for nodes greater than 50% are shown in the tree. The scale bar indicates 0.05 substitution per site.

Phylogenetic approaches based on Maximum Likelihood (ML) method (Fig 1) and Neighbor-Joining (NJ) method (Fig S1) were undertaken to get a clearer understanding of the grouping of the fungal sequences generated from this study with published cultured fungal and uncultured fungal-like 18S rRNA sequences.

The fungal clone type RH1 clustered in a distinct group within a larger Chaetothyriales clade (94% and 95% bootstrap support in ML and NJ methods); some taxa within this group were also represented by marine genera. The fungal clone type RH2 was recovered with the *Verticillium* sequences in a distinct clade that consisted of some other *Verticillium*-like species as well as with an uncultured fungal sequence with a marine origin. These were linked to, but distinct from, a group formed largely of species that were pathogens of other fungi. In both the phylogenetic methods the bootstrap support was very strong for this clade. Both RH1 and RH2 belong to the phylum Ascomycota based on the phylogeny.

Clone type RH3, along with deep-sea fungal 18S rRNA sequence types DS1 and DS7, were recovered in a distinct clade containing several uncultured fungal 18S rRNA sequences of diverse marine origin including amplicons from the gut of an amphipod from Pacific Ocean and anoxic sedimentary environment in the Arabian Sea (see Fig 1 and Fig S1). This clade belongs to the order Urotiales under the phylum Basidiomycota. Deep-sea fungal sequence types DS2 and DS8 formed a sub-cluster with the cultured fungus *Basidioradulum radula* of the order Hymenochaetales and an uncultured Agaromycotina clone belonging to the phylum Basidiomycota in ML and NJ trees. Deep-sea fungal sequence type DS3 clustered with cultured fungal genera belonging to the order Xylariales under the phylum Ascomycota with significant bootstrap support. The deep-sea fungal sequence types DS4 and DS5 were placed into two separate clades containing cultured and multiple uncultured fungi of deep-sea origin (Puerto Rico Trench, 6000 m depth) of the order Tremellales and Ustilaginales respectively under the phylum Basidiomycota, bootstrap support was significant for DS4 and DS5 in ML tree. The remaining fungal sequence type DS6 formed a cluster with cultured members and uncultured fungal sequences in the order Botryosphaeriales under the phylum Ascomycota.

The top matching OCTUs from environmental pyrosequencing datasets were further scrutinized for biogeographic patterns; the geographic distribution of sequence reads within each fungal OCTU reveals broad distributions across marine sample sites (Table S1). Two OCTUs (7349_B, top match to DS6; 8013_A, top match to RH3/DS1) appeared in both shallow water and deep-sea samples, pointing towards putatively cosmopolitan distributions for these groups.

## Discussion

### Methodological implications for nematode studies

The frequent detection of fungal 18S rRNA sequences along with nematodes from an offshore UK site and from a deep-sea site in the Southern Indian Ocean indicates that the commonly used nematode 18S primers are not nematode-specific. This has been confirmed in pyrosequencing investigations, which have successfully utilized one of our nematode primer sets (SSU_F04/SSU_R22) to amplify a wide range of eukaryotic phyla from environmental samples [25]. Primer binding sites for the 28S rRNA gene additionally appear to be quite conserved across phyla; fungal 28S rRNA-like sequences were also amplified from deep-sea specimens when using nematode primers targeting the D2/D3 expansion region (data not shown). This broad amplification range of common ribosomal primer sets has recently led to the development of improved nematode 18S primers [26].

For deep-sea nematodes, fungal sequences were most often obtained after specimens had been stored in slide mounts for long periods of time. Subsequent methodological investigations revealed that the slide mounting process can effectively degrade nematode DNA during storage [27], allowing instead for fungal amplicons to dominate PCR reactions. Generally, if nematode DNA is well-preserved when extracted, the signal from any fungal PCR amplicons is silenced by the overwhelming majority fraction of nematode PCR amplicons during protocols for direct sequencing. However, fungal DNA sequences can occasionally be obtained from specimens stored in slides for only a few hours, even when all precautions have been made to ensure the integrity of nematode DNA (H.Bik, unpublished data). In addition, fungal isolates from all nematode specimens showed a high pairwise identity (>95%) to environmental sequence data obtained via high-throughput sequencing of unpreserved (frozen) marine sediment. Thus, we are confident that fungal phylotypes presented in this study were recovered due to ecological associations with nematode specimens (e.g. present in the cuticle, gut) and do not represent preservation artefacts or post-processing fungal growth.

Methods for extracting nematode genomic DNA are quite ‘dirty’ compared to protocols for larger organisms, where an animal's size may allow for DNA to be extracted from a subsample of clean tissue (e.g. muscle or whole appendages). Lysis of single nematodes involves the digestion of entire specimens—thus nematode protocols inherently extract genomic DNA from all other organisms present in the nematode digestive tract and on the cuticle. Given the low amount of genomic material usually obtained from single nematodes, it is likely that fungal DNA and nematode DNA can act as competitive templates for primer binding during PCR reactions.

### Evidence for nematode-fungal associations

Frequent co-amplification of fungus and nematode sequences implies a close relationship between these two taxa; however, the precise association between fungal species and marine nematodes is presently unclear. Fungal sequences obtained from individual nematode specimens may represent gut contents, detritus attached to the cuticle, or alternatively, fungal species that exhibit symbiotic or pathogenic associations with nematodes. Fungal-feeding behaviours are well documented in soil nematodes [28] but there is little information regarding the feeding habitats of marine species; since nematodes and fungi are concurrently ubiquitous in coastal and deep-sea environments, fungi may play an important role in macrofaunal and meiofaunal food webs. In phylogenetic topologies (Fig 1), fungal clone RH1 is recovered within a group of taxa loosely referred to as ‘black yeasts’; this phylotype is most likely a member of the family Herpotrichiellaceae. These taxa are commonly isolated from a wide range of environmental samples, are known from European marine habitats [29], and are often either saprophytic or pathogenic [30] and some species can cause mycotic infections in humans and other animals. Other species such as *Exophiala castellanii* (*syn. Rhinocladiella mansonii*) have been isolated from nematode eggs [31]. This observed phylogenetic placement of clone RH1 may imply either a coincidental co-amplification of fungal and nematode sequences or a pathogenic relationship between nematodes and this fungal species [30].

Fungal associations have been recorded in several eukaryotic phyla, and thus evidence for relationships with nematodes is not entirely unexpected. Several studies have reported the presence of endophytic mycophycobionts associated with brown seaweeds [3], [32]; there are further reports of fungal associations with other marine algae and halophytes, with the majority of these fungi belonging to either Ascomycota or Deuteromycota [33], [34], [13]. Long-term fungal associations were also detected amongst healthy and decaying brown seaweed *Fucus serratus* thallii using the DGGE approach, with most isolates showing similarity to *Lulworthia*, *Lindra*, *Sigmoidea*/*Corollospora*, *Acremonium*/*Emericellopsis* and other ribotypes [34]. In our study we encountered several of our deep-sea fungal 18S rRNA sequences closely affiliated with uncultured and cultured fungal sequences belonging to Ascomycota and Basidiomycota based on BLAST results and confirmed in ML and NJ tree topologies. Previous studies have shown that members of Ascomycota and Basidiomycota dominate in the deep-sea environment along depth gradients [7]; similar observations were noted in the Puerto Rico trench, with taxonomic assessment suggesting that most fungal sequences were closely associated with parasitic or pathogenic species, a recurring finding in recent deep-ocean eukaryotic surveys [7], [35].

Both RH1 and RH2 fungal sequence types show striking similarity with fungal sequences detected previously from the coastal waters of southwest England using a PCR-DGGE approach [15]. Moreover, the detection of these fungal sequences in selected nematode 18S rRNA amplicons of *Terschellingia* sp., *Terschellingia longicaudata*, *Parodontophora* sp., *Viscosia viscosa, Sabatieria pulchra*, *Daptonema* sp. and *D. normandicum* suggests that the fungal sequence types (and the original fungi) may be restricted to certain group of nematodes; our results also correspond with nematode taxa that were observed to have co-amplified fungal sequences in a previous study [15].

### Putatively cosmopolitan phylotypes

The 18S rRNA is relatively conserved in fungi and has been used to reconstruct deep evolutionary relationships in cross-phyla phylogenetic studies [36]. Fungal 18S rRNA sequences are often used to identify orders and families in fungal molecular diversity studies [30], [37], although variation in this gene has been used to discriminate down to the level of genus and species [38]. In this study, fungal sequences exhibited high identity scores with GenBank and EMBL sequences, and the top BLAST matches for any given fungal clone were comprised of closely related fungal genera. These GenBank/EMBL sequences often represented isolates from remote locations; for example, a fungal sequence phylotype (DS1) amplified from a deep-sea nematode *Southerniella* sp. showed 100% identity with an uncultured fungus clone FAS_91 18S (Acc No GU072590) sequenced previously from the oxygen depleted sediments of the Arabian Sea [39]. Similar patterns were observed amongst phylotypes RH2 and DS5, which also showed 99% identities to unidentified 18S rRNA fungal clones amplified from diverse marine origins ranging from coral reef sand to marine sponge. Within our dataset, phylotypes RH3 and DS1 showed 99% sequence identity despite being collected from disparate locations and spanning a large bathymetric gradient. These phylotypes cluster together with the same OCTU (8013_A) representing the closest relative in an independent, high-throughput 18S dataset; this OCTU was observed in almost all environmental samples analyzed, representing deep-sea sites in two ocean basins (Pacific and Atlantic) and a shallow water site in Baja, CA. A similar ubiquitous pattern was observed for another OCTU (7349_B, the top BLAST hit for phylotype DS6), while other environmental OCTU matches appeared to be present in disparate deep-sea sites (Table S1). Regardless of the methodology, molecular data appears to consistently recover similar biogeographic patterns for marine fungi.

While we cannot confirm that highly similar fungal phylotypes represent the same biological species (given the conserved nature of 18S rRNA), our results suggest some intriguing patterns amongst marine fungi. Phylotypes exhibiting 100% pairwise identity to published sequences from remote locations may indeed represent broad geographic distributions for some fungal species; further work (including population genetic studies) will be required to confirm this hypothesis. In addition to geographic distance, these results suggest that some fungal species may adapt to different biogeochemical conditions (e.g. typical abyssal habitats and oxygen minimum zones). In an evolutionary context, highly similar sequences recovered from geographically disparate locations suggest (at minimum) closely related fungal species complexes that are globally distributed across marine environments.

Morphological evidence has long suggested cosmopolitan distributions for some marine fungi [40], although molecular data is now revealing widespread cryptic speciation and geographically structured populations in many fungi species [41]. It appears that fungal morphology (like nematode morphology) does not accurately reflect genetic differentiation amongst closely related species. However, our results suggest a contrary situation for at least some fungal taxa. The recovery of identical and highly similar fungal sequences from UK and Sub-Antarctic sediments (with further confirmation from 454 data), implies that at least some marine fungal populations may be conspecific over large geographic distances [29] with potentially cosmpolitan distributions.

The results from this investigation provide further evidence to support the existence of nematode-fungal associations in marine ecosystems. The recovery of identical or highly similar sequences in BLAST searches, coupled with geographic insight from 454 data, suggests that some fungal phylotypes are widespread and maintain cosmopolitan distributions. Despite these insights, a more intensive focus is needed to validate and quantify these putatively cosmopolitan fungal species. We currently do not understand the scope of nematode-fungal interactions, but limited data suggests a taxonomic bias for nematodes maintaining fungal associations. The ubiquity of both nematodes and fungi in benthic marine habitats suggests that both these taxa (and their interspecific relationships) fill key roles within these ecosystems. Finally, the present study highlights the utility of large environmental datasets for addressing specific biological hypotheses; high-throughput data generated in an independent study ultimately provided key insight towards elucidating the global distribution of marine fungal taxa. As sequencing costs continue to fall and more large datasets are generated, such datasets will likely play key roles in our understanding of global biodiversity.

## Materials and Methods

Fungal sequences were obtained during two independent investigations of nematode fauna: one study focusing on shallow water specimens collected off the UK Coast, and a second study investigating deep-sea taxa in the Southern Indian Ocean. Shallow-water sediments were collected off Rame Head (RH) (50m depth) (50° 17′ N, 4° 17′ W), an offshore site close to Plymouth in South West England in 2004 and were immediately fixed in molecular grade ethanol for further studies. Deep-sea sediments were collected aboard RRS Discovery cruise D300 in December 2005, with the sample location representing an abyssal plain site in the Southern Indian Ocean (near the Crozet Islands). Deep-sea samples were obtained using a megacorer (4232 m depth, 49° 3′ 38" S, 51° 14′ 12" E). Upon collection, the top 0-1 centimetre sediment fraction of each sample was immediately fixed in DESS preservative containing 5% DMSO. Nematodes analysed in this study were collected from site M6 of the CROZEX investigation; this particular sample area receives minimal organic flux from surface waters [16].

### Nematode extraction

Nematodes were extracted from sediments via decantation and flotation in Ludox, following standard methods [17]. A 45 µm sieve was required to process deep-sea samples, due to the small size of nematodes living in abyssal environments. Nematodes were removed from each sample under a low-power dissection microscope, using a fine wire tool. Specimens were transferred into dehydrating solution (water, glycerol, and molecular grade ethanol) and placed into a desiccator overnight. Nematodes were subsequently mounted in a drop of anhydrous glycerol on glass slides sealed with a wax ring. All specimens were examined under a compound microscope and identified based on standard taxonomic methods. Following identification, nematodes were individually transferred to 0.5 ml PCR tubes containing 20 µL of 0.25 M NaOH for DNA extraction. Variation in extraction, PCR and sequencing protocols is a reflection of different techniques utilized in the two independent labs where data was collected during this study.

### Genomic extractions from single nematodes

DNA was extracted following established protocol [18]. Deep-sea nematode extractions were modified slightly to exclude overnight incubation step at 60°C. Prior to PCR amplification, selected genomic extracts of deep-sea nematodes were amplified using the GenomiPhi V2 DNA Amplification kit (GE Healthcare UK Ltd, Little Chalfont, England) following manufacturer's instructions. Aliquots of the completed GenomiPhi reactions were subsequently used as template for PCR reactions.

### PCR amplification of the partial 18S rRNA

Nematode 18S rRNA primers from previous studies were used in this study [19], [20]. Four set of primers were used for PCR amplification which are as follows:

(i) SSU_F04 (5′-GCTTGTCTCAAAGATTAAGCC-3′) and SSU_R22 (5′-GCCTGCTGCCTTCCTTGGA-3′)

(ii) SSU_F22 (5′-TCCAAGGAAGGCAGCAGGC-3′) and SSU_R24 (5′-CCCCRRTCCAAGAATTTCACCTC-3′)

(iii) SSU_F22 (5′-TCCAAGGAAGGCAGCAGGC-3′) and SSU_R26 (5′-CATTCTTGGCAAATGCTTTCG-3′)

(iv) SSU_F24 (5′-AGAGGTGAAATTCTTGGATC-3′) and SSU_R13 (5′-GGGCATCACAGACCTGTTA-3′)

The lengths of the amplicons were 400 bp, 476 bp, 517 bp and 570 bp, respectively. For PCR parameters see published protocol [20]. PCR products were visualized on a 1% agarose gel containing ethidium bromide prior to cloning and DNA sequencing. Positive and negative PCR controls were used to confirm PCR success and check for potential airborne fungal contaminants within the laboratory environment.

### Cloning and DNA sequencing

PCR products from UK specimens were gel purified using a Qiagen Gel purification kit following manufacturer's instructions. These products were subsequently cloned using the pGEM-T Easy vector system (Promega Inc, Madison, USA). Plasmid DNA containing the inserts were cycle sequenced using BigDye Terminator Kit v3.1 (Applied Biosystems, Foster City, USA). Cycle sequencing reactions were cleaned using the Wizard Magnesil™ system (Promega Inc, Madison, USA). PCR products from deep-sea samples were cleaned using a QIAquick PCR purification kit (Qiagen), and cycle sequenced using a BigDye Terminator kit v3.1. Products from cycle-sequence reactions were cleaned via ethanol precipitation before being submitted for direct sequencing. For all specimens, sequencing was carried out in both directions using standard primers in an ABI Hitachi 3100 Genetic Analyzer. Generated sequences were then compared with known 18S rRNA sequences from GenBank and EMBL using the BLAST search engine (BLASTn program).

### Phylogenetic analysis

Sequences generated in this study were inspected closely to ensure that there were no PCR chimera events using CHIMERA-CHECK program [21]. Uncultured environmental and cultured 18S rRNA fungal sequences that overlapped with our sequences blast on BLAST validation and representatives of cultured fungal 18S rRNA diversity were included for alignment online at SILVA databases (http://www.arb-silva.de). The alignment was trimmed to the 5′end part of the fungal fragments (alignment length-415 bp) and was subsequently used for phylogenetic analysis. Phylogenetic tree based on maximum-likelihood (ML) with GTR+G+I model and gamma distribution was constructed in MEGA v5.0 [22]. The discrete gamma + invariable sites model accommodates site to site variability of evolutionary rate and an estimated proportion of invariant sites and was chosen for the ML compared to F84 model. For the neighbor-joining method (NJ) Kimura-2-parameter and gamma distribution was the basis for phylogenetic tree construction in MEGA v5.0 [22]. The tree was subsequently bootstrapped with 1000 replicates. The 18S rRNA sequence of *Rhizomucor miehei* (Acc No AF192506) (member of Fungi incertae sedis) was used as outgroup in both the trees. Environmental sequences reported in this paper have been submitted to GenBank and their accession numbers are from JF708047-JF708073.

### Comparative 454 datasets

Environmental pyrosequencing datasets (18S rRNA amplicon libraries obtained with SSU_F04/SSU_R22 tagged primers [23]) were previously obtained from marine sediments representing shallow water and deep-sea sites in both Atlantic and Pacific oceans (Table S2). Fungal phylotypes obtained in this study were compared to Operationally Clustered Taxonomic Units (OCTUs) constructed from raw 454 pyrosequencing reads (quality checked and filtered) clustered under a 99% pairwise identity cutoff in the OCTUPUS pipeline [24]. Fungal sequences were compared against non-chimeric OCTU consensus sequences using BLASTn; the top OCTU hit was taken to represent the closest relative in 454 datasets. Consensus sequences from each matching OCTU have been included as File S1. The pyrosequencing datasets reported in this paper will be archived in Dryad (http://datadryad.org/).

## Supporting Information

Figure S1 Neighbor Joining tree with bootstrap values constructed using fungal 18S rRNA sequences generated from this study and corresponding BLAST matches with highly identity scores recovered from GenBank and EMBL and published cultured fungal 18S rRNA sequences. Bootstrap values for nodes greater than 50% are shown in the tree. The scale bar indicates 0.05 substitution per site.
(TIF)

Table S1 Geographic distribution of sequence reads contained within each top-scoring environmental OCTU, including number of raw sequences within each cluster.
(DOC)

Table S2 Location information for sites analysed as part of an independent 454 study of marine habitats.
(DOCX)

File S1 FASTA file containing OCTU consensus sequences of the top-scoring BLAST hits in an environmental 454 dataset.
(TXT)
