# Supplementary figures and images for: Molecular Diversity of Fungal Phylotypes Co-Amplified Alongside Nematodes from Coastal and Deep-Sea Marine Environments
Source: PLoS One. 2011 Oct 26;6(10):e26445. doi: 10.1371/journal.pone.0026445 (PMC3202548; doi:10.1371/journal.pone.0026445)

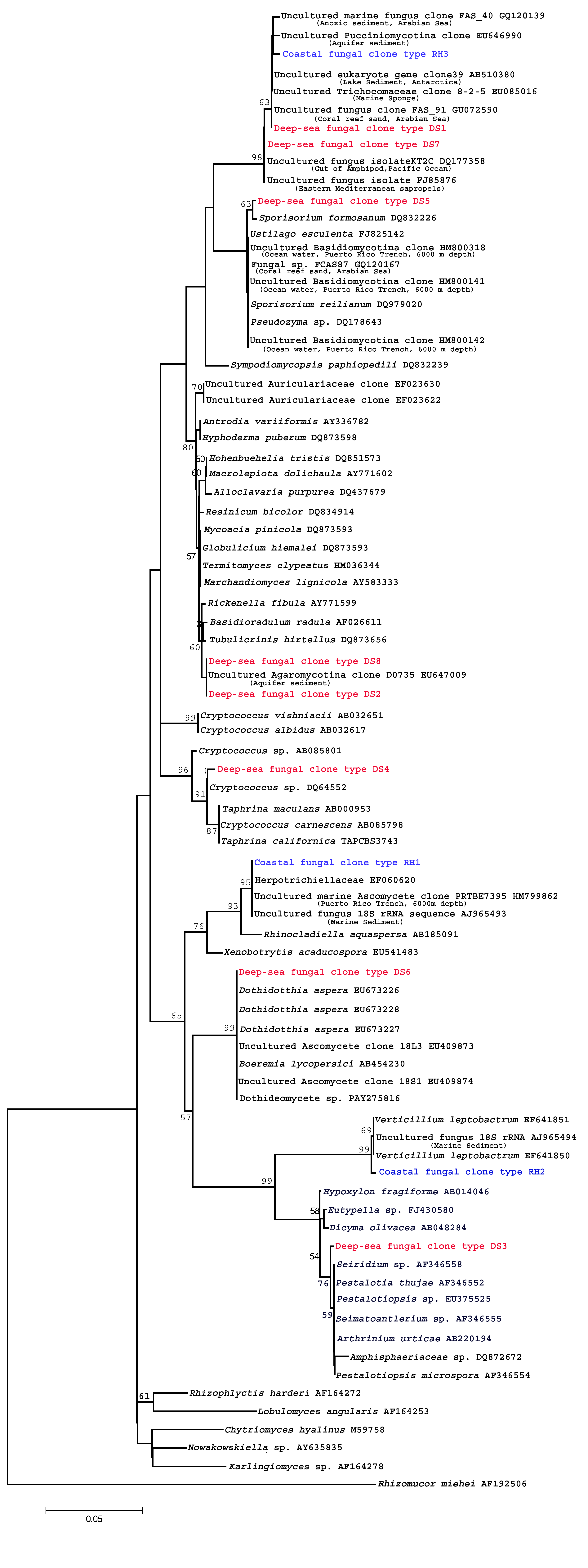

Supplement: Figure S1 — Neighbor Joining tree with bootstrap values constructed using fungal 18S rRNA sequences generated from this study and corresponding BLAST matches with highly identity scores recovered from GenBank and EMBL and published cultured fungal 18S rRNA sequences. Bootstrap values for nodes greater than 50% are shown in the tree. The scale bar indicates 0.05 substitution per site. (TIF) [file pone.0026445.s001.tif]
