# Supplementary material for: Molecular Diversity of Fungal Phylotypes Co-Amplified Alongside Nematodes from Coastal and Deep-Sea Marine Environments
Source: PLoS One. 2011 Oct 26;6(10):e26445. doi: 10.1371/journal.pone.0026445 (PMC3202548; doi:10.1371/journal.pone.0026445)
Supplement: Table S1 — Geographic distribution of sequence reads contained within each top-scoring environmental OCTU, including number of raw sequences within each cluster. (DOC) [file pone.0026445.s002.doc]

| SSU_F04 Primer | | | SSU_R22 Primer | | |
| --- | --- | --- | --- | --- | --- |
| 128 237 321 422 528 | 22#1 29 43 45 | SF | 128 237 321 422 528 | 22#1 29 43 45 | SF |

Table S1

| **OCTU** |  |  |  |  |  |  |  |  |  |  |  |  |  |  |  |  |  |  |  |  | Total  Reads | # Sites |
| --- | --- | --- | --- | --- | --- | --- | --- | --- | --- | --- | --- | --- | --- | --- | --- | --- | --- | --- | --- | --- | --- | --- |
| 12052_B | 0 | 0 | 0 | 0 | 0 | 0 | 0 | 0 | 0 | 0 | 0 | 1 | 0 | 0 | 0 | 0 | 51 | 0 | 0 | 0 | 52 | 2 |
| 19989_B | 0 | 0 | 0 | 0 | 0 | 0 | 0 | 0 | 0 | 0 | 0 | 0 | 0 | 4 | 0 | 0 | 0 | 0 | 0 | 0 | 4 | 1 |
| 58922_B | 0 | 0 | 0 | 0 | 0 | 0 | 0 | 0 | 0 | 0 | 0 | 0 | 0 | 0 | 0 | 2 | 0 | 0 | 0 | 0 | 2 | 1 |
| 11214_B | 0 | 0 | 0 | 0 | 0 | 0 | 0 | 0 | 0 | 0 | 0 | 0 | 0 | 0 | 0 | 0 | 1 | 16 | 0 | 0 | 17 | 2 |
| 7349_B | 0 | 0 | 0 | 0 | 0 | 0 | 0 | 0 | 0 | 0 | 0 | 0 | 1 | 0 | 0 | 0 | 0 | 63 | 0 | 1 | 65 | 3 |
| 9964_B | 0 | 0 | 0 | 0 | 0 | 0 | 0 | 0 | 0 | 0 | 0 | 6 | 0 | 0 | 0 | 0 | 0 | 0 | 1 | 0 | 7 | 2 |
| 8013_A | 8 | 14 | 6 | 0 | 2 | 0 | 3 | 6 | 1 | 9 | 0 | 0 | 0 | 0 | 0 | 0 | 0 | 0 | 0 | 0 | 49 | 8 |
| 22018_A | 0 | 0 | 0 | 0 | 0 | 0 | 0 | 0 | 3 | 0 | 0 | 0 | 0 | 0 | 0 | 0 | 0 | 0 | 0 | 0 | 3 | 1 |
| 64386_A | 2 | 0 | 0 | 0 | 1 | 0 | 0 | 0 | 0 | 0 | 0 | 0 | 0 | 0 | 0 | 0 | 0 | 0 | 0 | 0 | 3 | 2 |
