# Supplementary material for: Molecular Diversity of Fungal Phylotypes Co-Amplified Alongside Nematodes from Coastal and Deep-Sea Marine Environments
Source: PLoS One. 2011 Oct 26;6(10):e26445. doi: 10.1371/journal.pone.0026445 (PMC3202548; doi:10.1371/journal.pone.0026445)
Supplement: Table S2 — Location information for sites analysed as part of an independent 454 study of marine habitats. (DOCX) [file pone.0026445.s003.docx]

|  | **Sediment**  **Type** | **Collection**  **Date** | **Latitude** | **Longitude** | **Depth** |
| --- | --- | --- | --- | --- | --- |
| **Pacific128** | Fine mud | 16-Sep-08 | 43° 59.971' N | 130° 23.531' W | 3200 m |
| **Pacific237** | Fine mud | 18-Sep-08 | 42° 33.588' N | 131° 55.040' W | 3585 m |
| **Pacific321** | Fine mud | 21-Sep-08 | 39° 59.400' N | 125° 52.406' W | 3664 m |
| **Pacific422** | Fine mud | 21-Sep-08 | 39° 59.695' N | 125° 26.385' W | 2712 m |
| **Pacific528** | Fine mud | 23-Sep-08 | 36° 48.024' N | 123° 41.774' W | 3678 m |
| **Atlantic22#1** | Fine mud | 15-Aug-08 | 35° 33.28' N | 9° 41.92' W | 4321 m |
| **Atlantic29** | Fine mud | 17-Aug-08 | 36° 13.06' N | 10° 01.82' W | 4878 m |
| **Atlantic43** | Fine mud | 22-Aug-08 | 38° 21.66' N | 9° 59.08' W | 4572 m |
| **Atlantic45** | Fine mud | 23-Aug-08 | 38° 23.18' N | 10° 24.13' W | 4835 m |
| **SF (Baja, CA)** | Medium sand | 4-Jul-08 | 31º 1.509' N | 114º 49.912' W | intertidal |

**Table S2**
